# Identification of a potent antagonist of smoothened in hedgehog signaling

**DOI:** 10.1186/s13578-021-00558-9

**Published:** 2021-03-02

**Authors:** Junwan Fan, Haowen Li, Lun Kuang, Zichen Zhao, Wenyan He, Chen Liu, Yongjun Wang, Steven Y. Cheng, Wei Chen

**Affiliations:** 1 China National Clinical Research Center for Neurological Diseases, Beijing Tiantan Hospital, Capital Medical University, Beijing, 100070 China; 2 Beijing Key Laboratory of Translational Medicine for Cerebrovascular Disease, Beijing, 100070 China; 3 Department of Developmental Genetics, School of Basic Medical Sciences, Nanjing Medical University, Nanjing, 210000 Jiangsu China

**Keywords:** 0025A, Antagonist, Cancer, Hedgehog signaling, Primary cilia, Smoothened mutation

## Abstract

**Background:**

Hedgehog signaling is essential to the regulation of embryonic development, tissue homeostasis, and stem cell self-renewal, making it a prime target for developing cancer therapeutics. Given the close link between aberrant Hedgehog signaling and cancers, many small molecular compounds have been developed to inhibit Smoothened, a key signal transducer of this pathway, for treating cancer and several such compounds have been approved by the United States Food and Drug Administration (GDC-0449 and LDE-225). However, acquired drug resistance has emerged as an important obstacle to the effective use of these first generation Hedgehog pathway blockers. Thus, new Smoothened inhibitors that can overcome such resistance is an urgent need going forward.

**Results:**

We established the Smoothened/βarrestin2-GFP high-throughput screening platform based on the mechanistic discovery of Hedgehog signaling pathway, and discovered several active small molecules targeting Smoothened including 0025A. Here we show that 0025A can block the translocation of βarrestin2-GFP to Smoothened, displace Bodipy-cyclopamine binding to wild-type Smoothened or mutant Smoothened-D473H, reduce the accumulation of Smo on primary cilia and the expression of Gli upon Hedgehog stimulation. In addition, we show that 0025A can effectively suppress hair follicle morphogenesis and hair growth in mice.

**Conclusions:**

Our results demonstrate that 0025A is a potent antagonist targeting Smoothened wild-type and mutant receptors in the Hedgehog signaling pathway and may provide a new therapy for refractory cancers.

## Background

Originally discovered in Drosophila *melanogaster*, the evolutionarily conserved Hedgehog (Hh) signaling pathway plays critical roles in promoting embryonic development [1, 2]. Upon completing embryogenesis, this pathway mostly becomes dormant except in certain tissues and under specific conditions such as during hair follicle morphogenesis and tissue repair [3, 4]. It has reported that Hh signaling is essential for regulating growth and morphogenesis of hair follicle [5].

Currently, it is known that the transduction of Hh signaling is regulated through Ptch1-Smo-Gli1 [6–8]. In the absence of Hh ligands, the twelve-transmembrane receptor Patched1 (Ptch1) suppresses the activity of seven-transmembrane receptor Smoothened (Smo), which is homologous to the G-protein coupled receptors, and leads to negatively regulate the Hh signaling pathway. However, in the presence of Hh ligands, Hh ligands directly bind to Ptch1 and alleviate its inhibition of Smo [9–11]. As a consequence, the activated Smo promotes Gli transcription factors to translocate into nucleus and regulates target genes expression.

Aberrant activation of Hh signaling pathway is related to the development of many cancers including basal cell carcinoma (BCC), medulloblastoma (MB) and many solid tumors [12, 13]. According to recent studies, PTCH1 mutation is the cause of basal cell nevus syndrome (also known as Gorlin syndrome), which significantly increases the risk of patients with BCC and MB [14, 15]. In addition, it has been reported that the inactivation mutation of PTCH1 and the activation mutation of Smo have been found in most spontaneous BCC and MB [16, 17]. Given the close relationship between excessive Hh signaling pathway and cancer, numerous researches have been devoted to design and synthesize small molecule inhibitors of Smo for treatment of cancers [18–20].

The transmembrane protein Smo is the main transducer of Hh signaling pathway and can be used as an important drug target [21, 22]. Thus far, there are many Smo inhibitors have been under investigation in clinical trials. Among them, GDC-0449 (vismodegib) was approved by FDA for treatment of advanced BCC in 2012 [23–25]. Unfortunately, a recurring case revealed the drug resistance to GDC-0449 due to Asp473 to His mutation (D473H) in Smo, which would suppress the interaction between drug and receptor [26, 27]. In addition, reports indicated that about 40% BCC patients acquired drug resistance carry the Smo mutations. Of these, 17% patients contained the Smo-D473H type [28]. Therefore, new Smo inhibitors that could inhibit wild-type and mutant receptors are essential for overcoming drug resistance.

Previous reports have demonstrated that β-arrestins are cytosolic adapter proteins, which would form complexes with most activated seven-transmembrane receptors after the receptors were phosphorylated by G-protein coupled receptor kinases [29]. Our preliminary results have shown that constitutively active wild-type Smo could recruit βarrestin2-GFP (βarr2-GFP) to the plasma membrane [10]. βarr2-GFP was evenly distributed in the cytoplasm when expressed alone in U2OS cells. Furthermore, co-expressing a chimeric form of Smo (Smo-633) swapping the C terminus of Smo with vasopressin type 2 receptor and βarr2-GFP in cells caused an obvious redistribution of βarr2-GFP to intracellular vesicles as aggregates, which could be blocked by Smo antagonist cyclopamine [10, 30, 31]. Based on these findings, we constructed the cell-based high-throughput screening assay and searched small molecule chemical libraries for potent Smo inhibitors by using this high-throughput assay. Here we show that several active hits including 0025A are discovered. It can block the translocation of βarr2-GFP to Smo, displace Bodipy-cyclopamine binding to wild-type Smo or mutant Smo-D473H, effectively reduce the accumulation of Smo on primary cilia and the expression of Gli upon Hh stimulation as well as suppress hair follicle morphogenesis and hair growth in mice. Our results demonstrate that 0025A is a potent antagonist targeting Smo receptors in the Hh signaling pathway and may provide a therapy for refractory cancers.

## Results

### Identification of compound 0025A

**Fig. 1:**
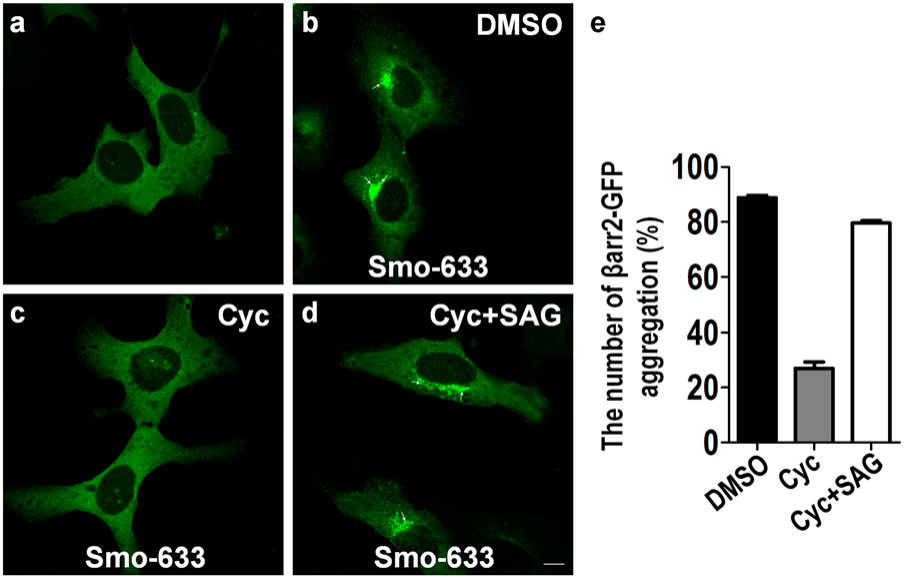
The translocation of βarr2-GFP to Smo in U2OS cells. The U2OS cells stably overexpressing βarr2-GFP alone (**a**) or βarr2-GFP and Smo-633 (**b-d**). Cells were treated with DMSO vehicle (**b**), 1 μM cyclopamine (Cyc) (**c**), or 1 μM Cyc and 1 μM SAG (**d**) for 24 h at 37 ℃. The arrows showed the intravesicular aggregation of βarr2-GFP. **e** Percentage of the number of βarr2-GFP aggregation was quantitated. Scale bar, 10 µm

**Fig. 2:**
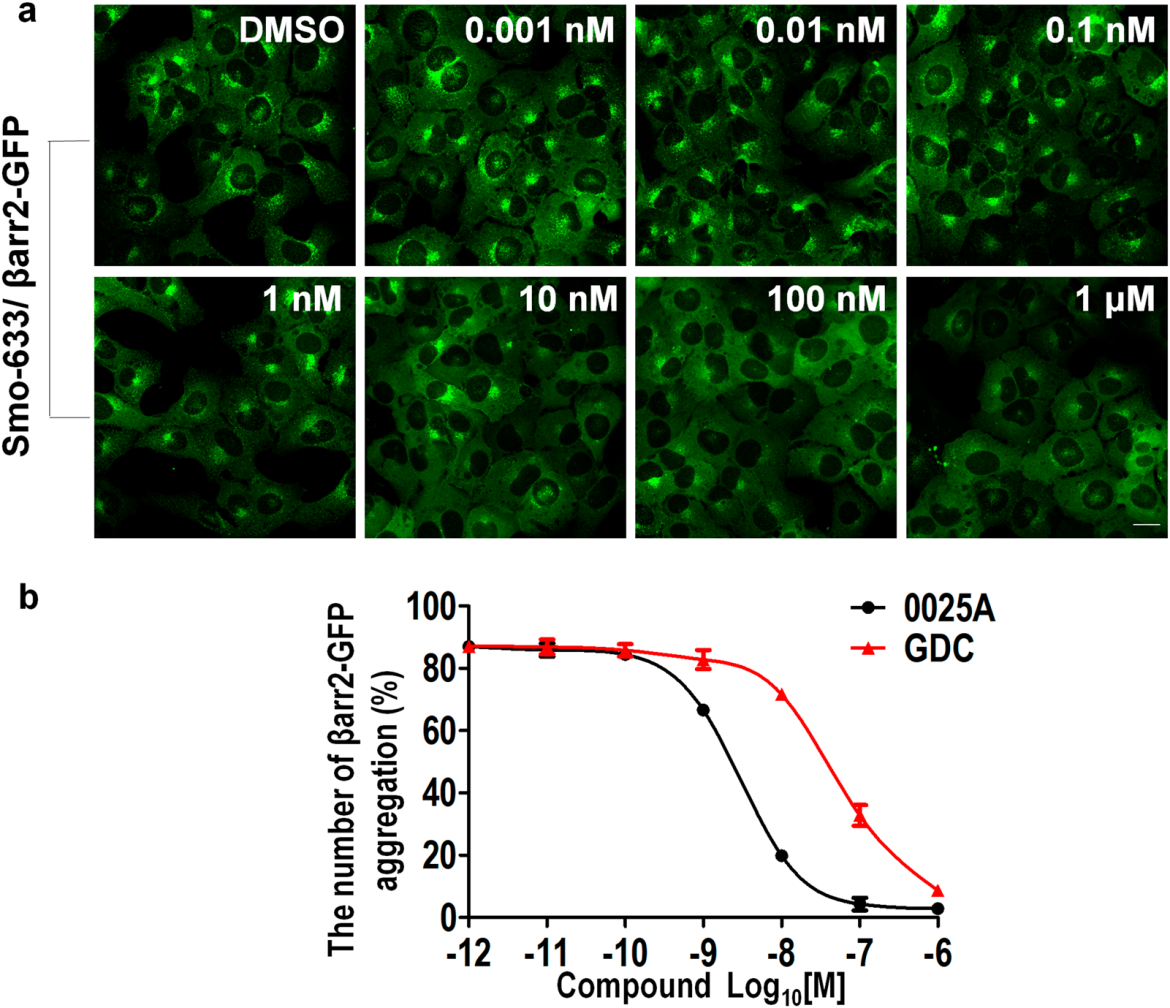
0025A inhibits the intravesicular aggregation of βarr2-GFP. **a** The U2OS cells stably overexpressing βarr2-GFP and Smo-633 were treated with different concentration of 0025A (10^–12^-10^–6^ M) for 24 h at 37 ℃. **b** Percentage of the number of βarr2-GFP aggregation was quantitated. GDC represented GDC-0449. Scale bar, 20 µm

**Fig. 3:**
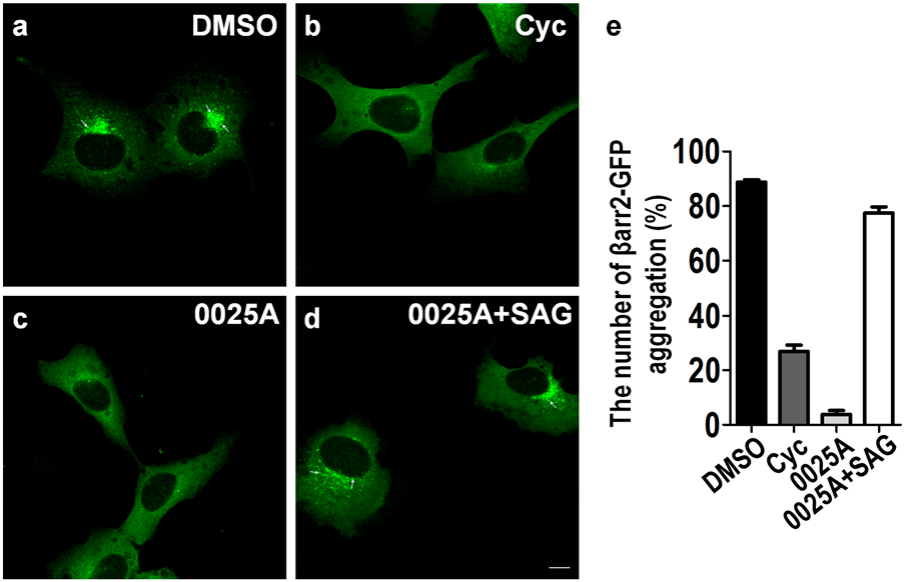
Identification of novel Smo inhibitors in U2OS cells. The U2OS cells stably overexpressing βarr2-GFP and Smo-633 were treated with DMSO (**a**), 1 μM cyclopamine (Cyc) (**b**), 100 nM 0025A (**c**), or 100 nM 0025A and 1 μM SAG (**d**) for 24 h at 37 ℃. The arrows showed the intravesicular aggregation of βarr2-GFP. **e** Percentage of the number of βarr2-GFP aggregation was quantitated. Scale bar, 10 µm

**Fig. 4:**
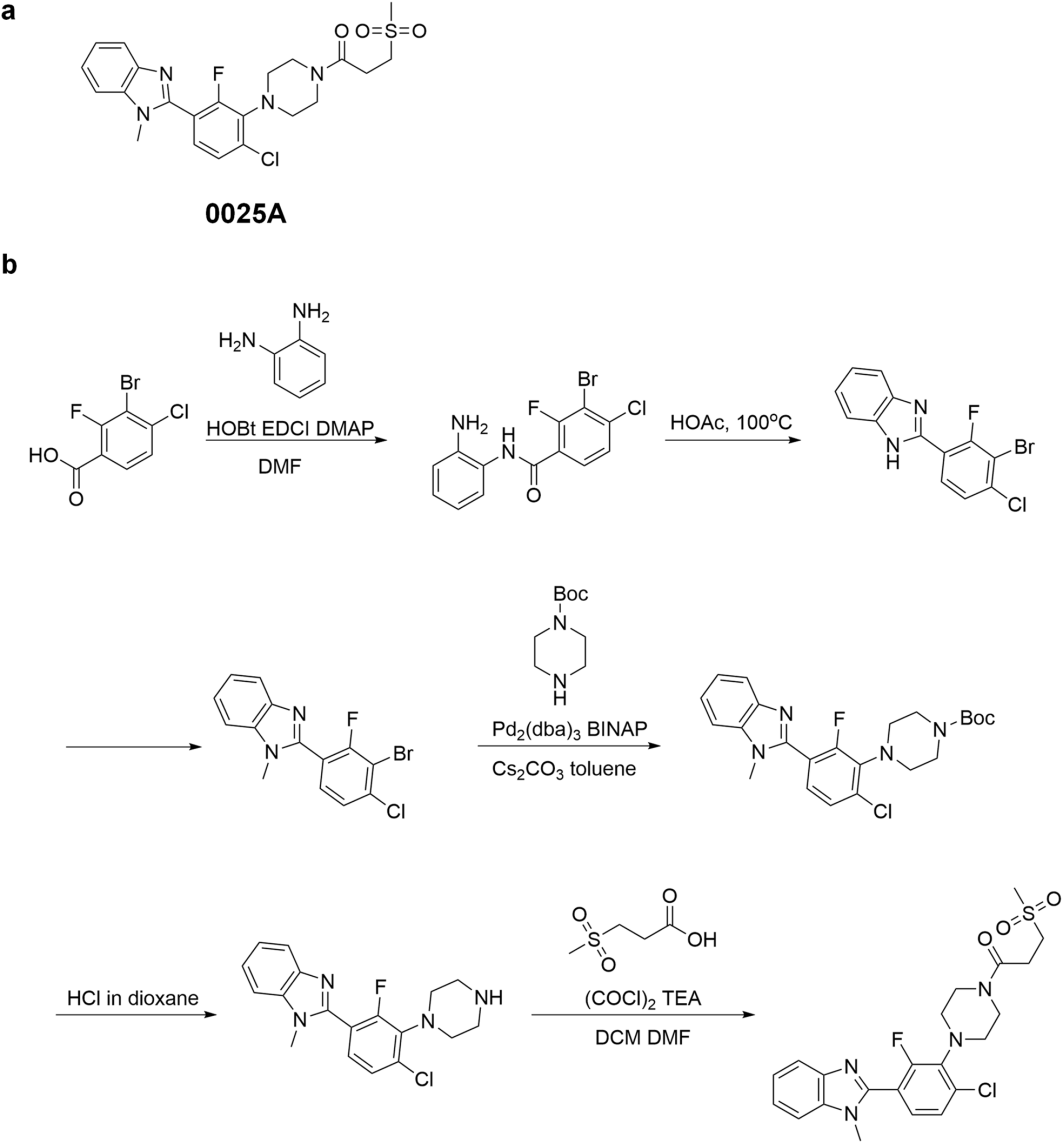
Chemical structure and synthesis of compound 0025A. **a** Structure of compound 0025A. **b** Synthesis of compound 0025A

As we showed previously, βarr2-GFP was distributed evenly in the cytoplasm when it was expressed alone in U2OS cells, which are commonly used for high-throughput cancer drug screens [31] (Fig. 1a). However, co-expressing βarr2-GFP with Smo-633 caused an ostensible redistribution of βarr2-GFP to intracellular aggregates/vesicles as evident by the appearance of bright aggregates surrounding the nuclei (Fig. 1b). Smo antagonist cyclopamine (Cyc) treatment led to the disappearance of green vesicles (Fig. 1c). Addition of Smo agonist SAG in the presence of Cyc led to the reappearance of aggregates of green vesicles (Fig. 1d). The images were quantitated in Fig. 1e. Based on this assay, we built up the primary high-throughput screening assay and screened our propriety small molecules library to identify small molecules that could prevent the-intravesicular aggregation of βarr2-GFP. We found that a lead compound 0025A could be able to inhibit the cellular puncta formation of βarr2-GFP (Fig. 2a). The IC_50_ of 0025A and GDC-0449 was 1.7 nM and 28 nM, respectively as determined by our screening assay (Fig. 2b). The activity of 0025A on Smo was further validated by Smo/βarr2-GFP internalization assay using Cyc and SAG as controls (Fig. 3). The structure of 0025A is shown in Fig. 4a. It was synthesized using the route described in Fig. 4b.

### 0025A is a competitive antagonist of Smo

**Fig. 5:**
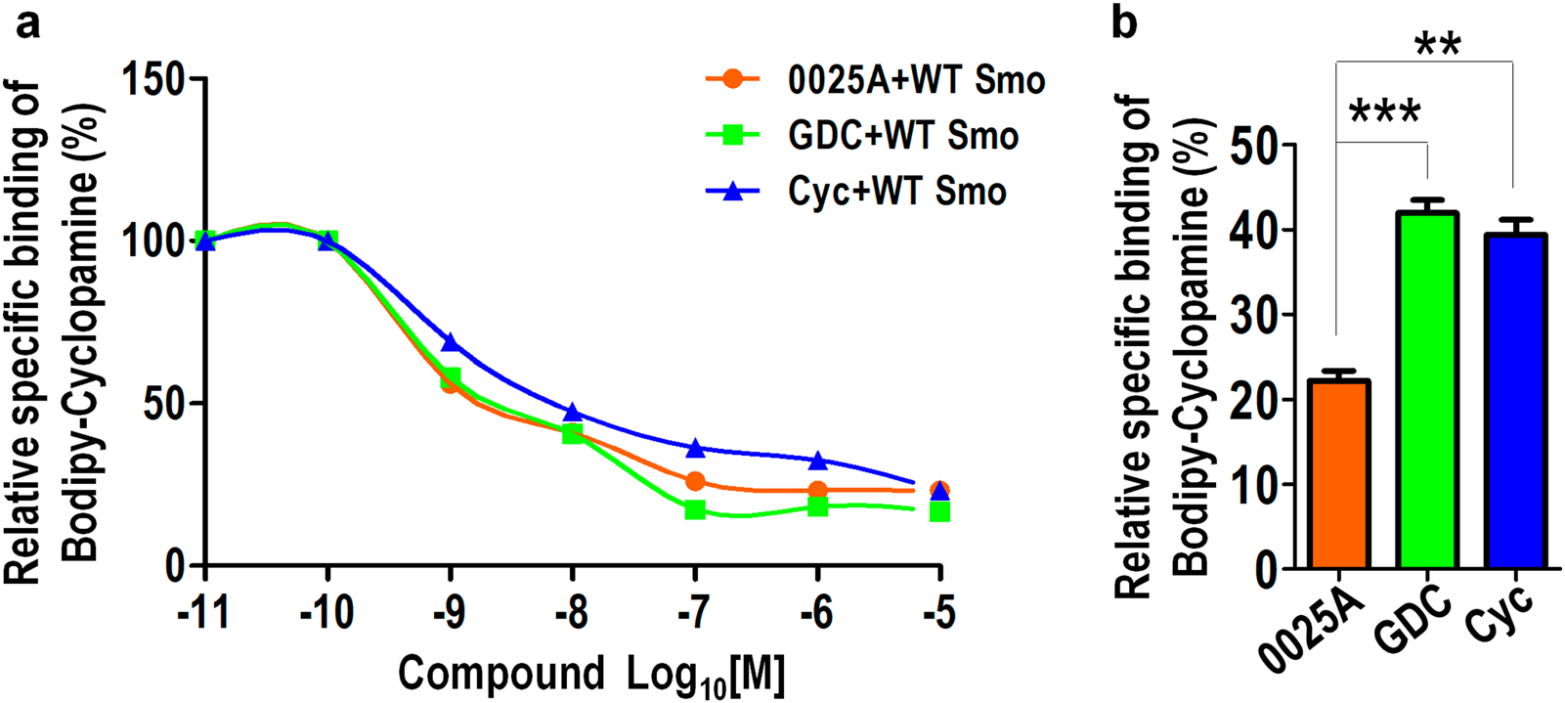
0025A competitively replaces Bodipy-cyclopamine binding to wild-type Smo and mutant Smo-D473H. **a** Competitive binding of Bodipy-cyclopamine to wild-type Smo with different concentration of Smo antagonists: 0025A, GDC-0449 (GDC), cyclopamine (Cyc). The Bodipy-cyclopamine competitive assay was performed in HEK293 cells transiently transfected with human wild-type Smo. **b** Competitive binding of Bodipy-cyclopamine to mutant Smo with 10^–6^ M (1 μM) Smo antagonists. The Bodipy-cyclopamine competitive assay was performed in HEK293 cells transiently transfected with human mutant Smo-D473H. The results of Bodipy-cyclopamine binding (green) were analyzed by flow-cytometry. All data are means ± SEM (student’s t-test). **P < 0.01, ***P < 0.001

To further verify the binding of 0025A to Smo, we performed the Bodipy-cyclopamine competition binding assay, which has been used to detect ligand binding to Smo [32]. We found that known Smo antagonists (cyclopamine, GDC-0449) and 0025A would be able to displace 5 nM Bodipy-cyclopamine from Smo with similar affinities (Fig. 5a). Recent studies have reported that a mutation of Smo (Smo-D473H) was resistance to GDC-0449 therapy [26]. To examine whether 0025A was able to bind with Smo-D473H, we overexpressed Smo-D473H in HEK293 cells and conducted the competition binding assay, which demonstrated that 0025A can effectively displace 5 nM Bodipy-cyclopamine from Smo-D473H. However, GDC-0449 was unable to bind with mutant Smo-D473H at low concentration and represented partial effect at high concentration consistent with previous reports [33, 34] (Fig. 5b). These results indicate that 0025A can compete with cyclopamine to bind with wild-type Smo and Smo-D473H receptor.

### Inhibition of Sonic hedgehog signaling

**Fig. 6:**
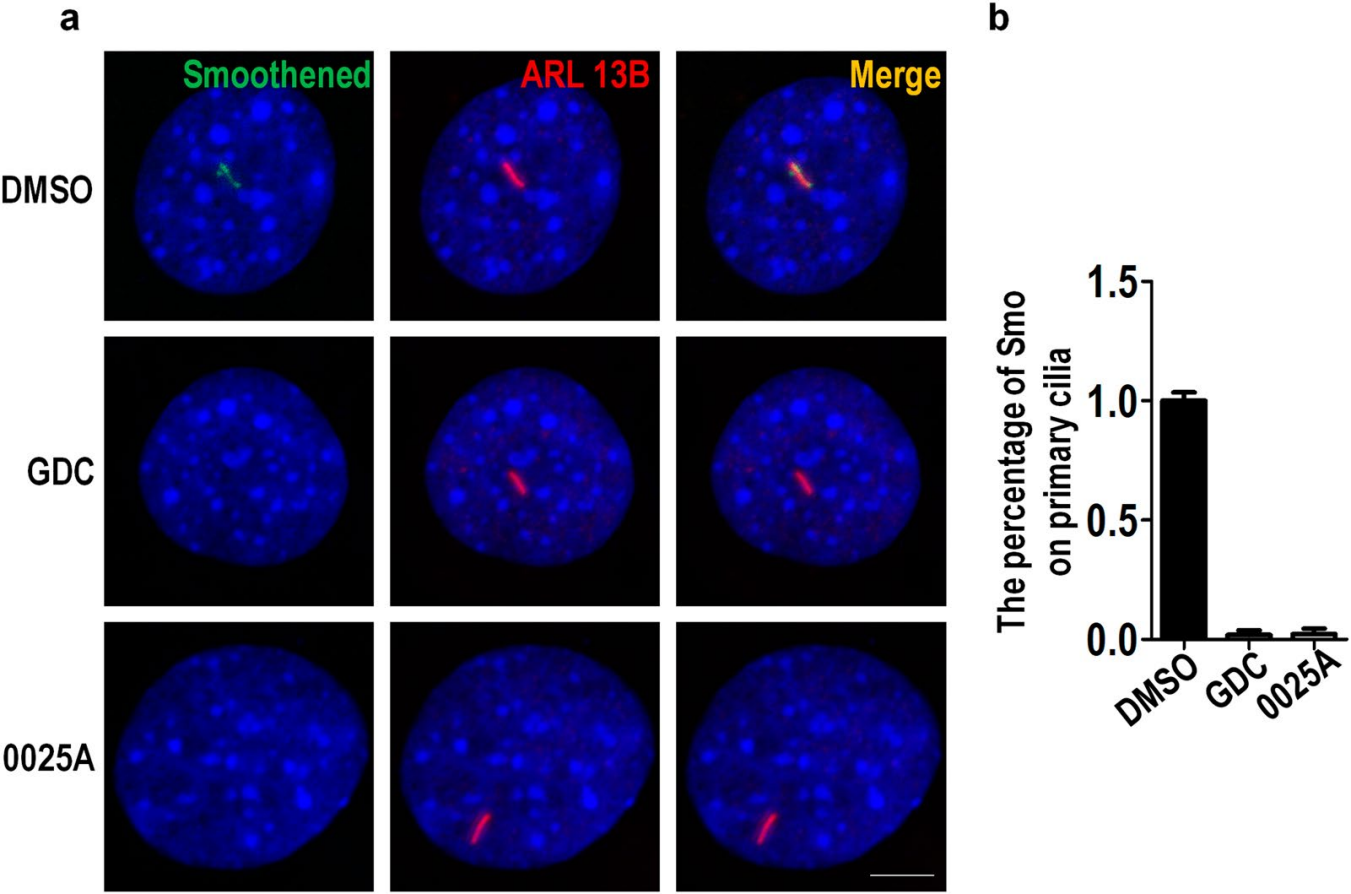
0025A inhibits the accumulation of Smo on primary cilia. **a** Serum-starved Ptch^−/−^ MEF cells were treated with DMSO, 10 μM GDC-0449 (GDC) and 10 μM 0025A for 24 h at 37 ℃. Then the cells stained with antibodies against Smoothened (Green) and ARL 13B (Red), a marker for primary cilia. **b** Percentage of Smo on primary cilia was quantitated (n = 100). Scale bar, 5 µm

We next tested the inhibitory effect of 0025A on Sonic hedgehog (Shh) signaling. Previous studies have reported that Smo mediates Shh signaling pathway by locating on primary cilia [35, 36]. To examine whether 0025A regulated the Smo primary cilia accumulation, we treated the Ptch^−/−^ MEF cells with DMSO, 10 μM GDC-0449, and 10 μM 0025A for 24 h, and immunostained the cells with antibodies against Smoothened and ARL 13B, a marker for primary cilia. We observed that compared to DMSO control, 0025A inhibited Smo primary cilia accumulation similar to the effect of the positive control GDC-0449 which could also suppress Smo primary cilia accumulation (Fig. 6a, b).

**Fig. 7:**
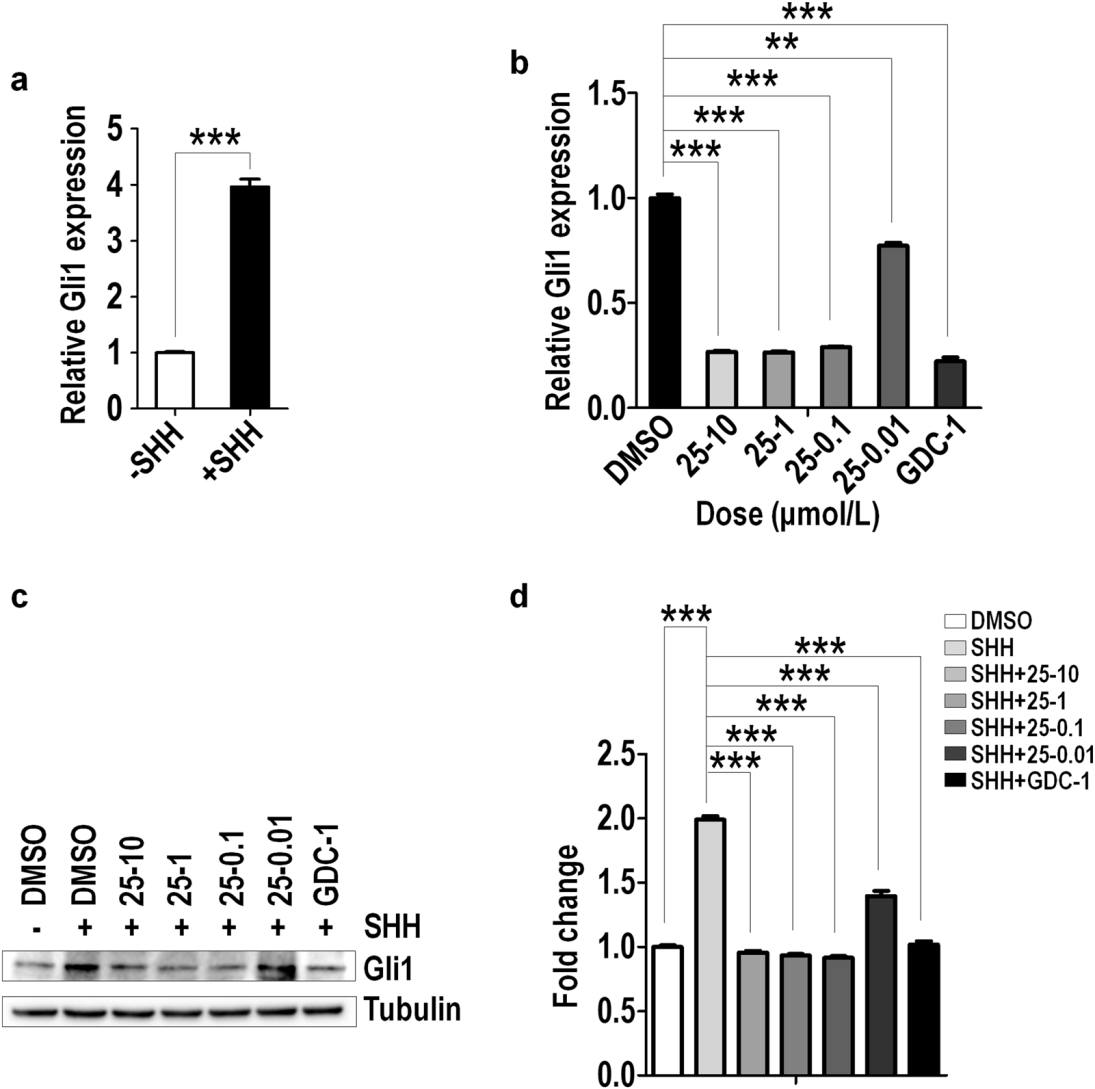
0025A inhibits the expression of Gli1 induced by Shh-CM. **a** Serum-starved NIH3T3 cells were treated with Shh-CM for 24 h. Cells were analyzed for mRNA levels of Gli1. **b-c** Serum-starved NIH3T3 cells were treated by Shh-CM with vehicle, different concentration of 0025A, or 1 μM GDC-0449 (GDC-1) for 24 h. Cells were analyzed for mRNA levels (**b**) and the protein levels (**c**) of Gli1. **d** Quantitation of Gli1 protein levels normalized to Tubulin loading control. All data are means ± SEM (student’s t-test). **P < 0.01, ***P < 0.001

**Fig. 8:**
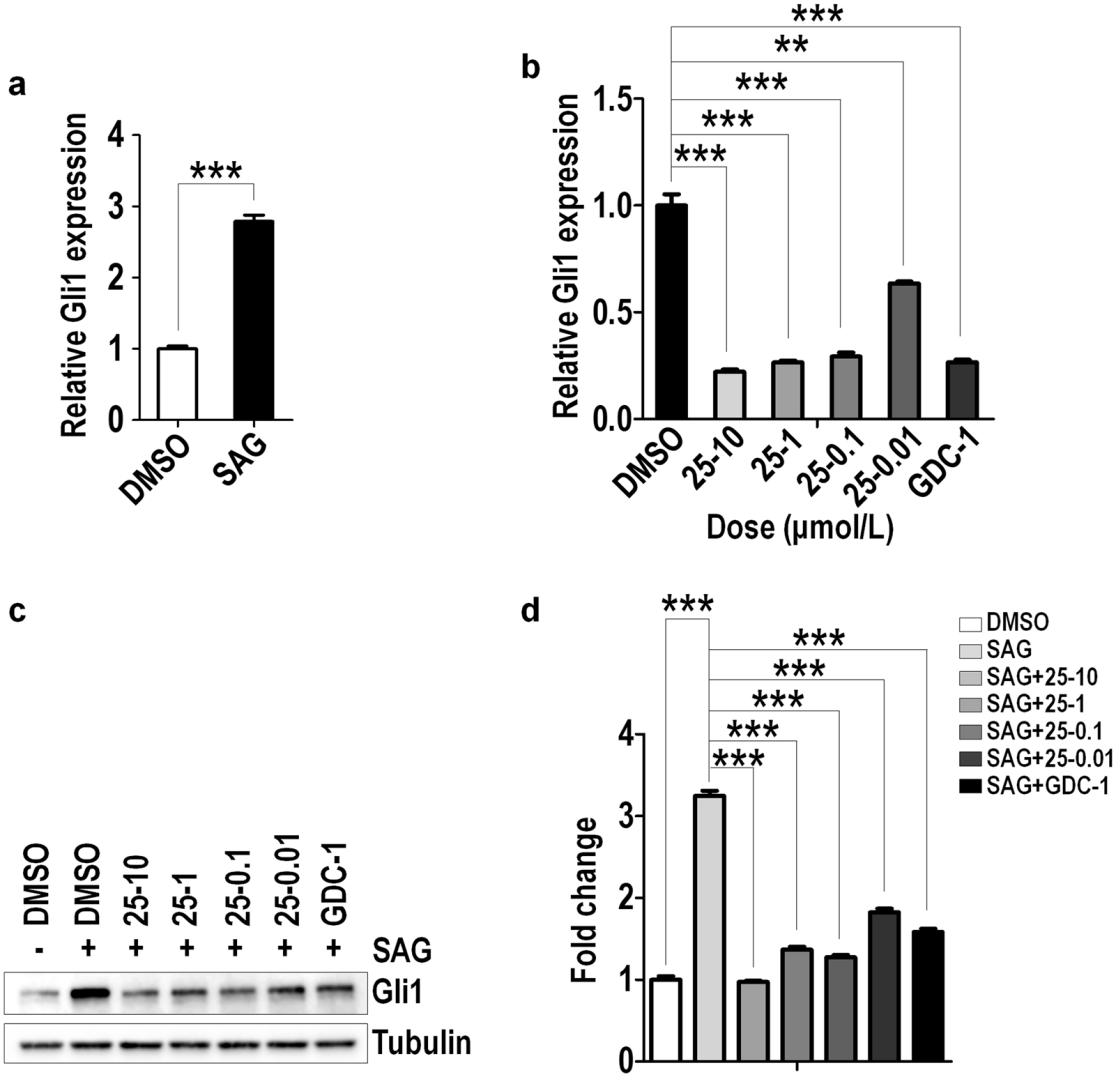
0025A inhibits the expression of Gli1 induced by SAG. **a** Serum-starved NIH3T3 cells were induced with DMSO or SAG for 24 h. Cells were analyzed for mRNA levels of Gli1. **b**, **c** Serum-starved NIH3T3 cells were induced by 10^–7^ M (100 nM) SAG with vehicle, different concentration of 0025A, or 1 μM GDC-0449 (GDC-1) for 24 h. Cells were analyzed for mRNA levels (**b**) and the protein levels (**c**) of Gli1. **d** Quantitation of Gli1 protein levels normalized to Tubulin loading control. All data are means ± SEM (student’s t-test). **P < 0.01, ***P < 0.001

It is known that Gli1 is the downstream target gene of Shh signaling, which serves as a readout of Shh signaling pathway activity [37, 38]. To verify the inhibition of 0025A on Shh signaling, we used *N-Shh* conditioned medium (Shh-CM) or a known Smo agonist SAG to enhance Gli1 transcription. We firstly prepared Shh-CM and explored the optimal effective concentration of SAG through a gradient concentration treatment, which is 100 nM. Then we stimulated the serum-starved NIH3T3 cells with 20% Shh-CM or 100 nM SAG, and found the mRNA level of Gli1 increased fourfold and threefold, respectively (Figs. 7a, 8a). To examine the inhibitory role of 0025A, we induced the serum-starved NIH3T3 cells by 20% Shh-CM with vehicle, 0025A (0.01, 0.1, 1, 10 μM) or GDC-0449 (1 μM) for 24 h, and observed that 0025A dose-dependently reduced the mRNA and protein level of Gli1. The positive control GDC-0449 could also suppress the expression of Gli1 (Fig. 7b–d).

Furthermore, we demonstrated that Smo agonist SAG stimulated Shh activitiy could be suppressed by 0025A using similar approaches as Shh-CM (Fig. 8b–d). In addition, we also detect the expression of Ptch1, which is another target gene of Hh signaling pathway [39], and found that 0025A could dose-dependently inhibit the mRNA level of Ptch1 induced by 20% Shh-CM (Additional file 1: Fig. S1). Taken together, the above results indicate that 0025A can act as a potent inhibitor and dose-dependently inhibit target gene expression of the Shh signaling pathway.

### 0025A inhibits hair regrowth and hair follicle morphogenesis

**Fig. 9:**
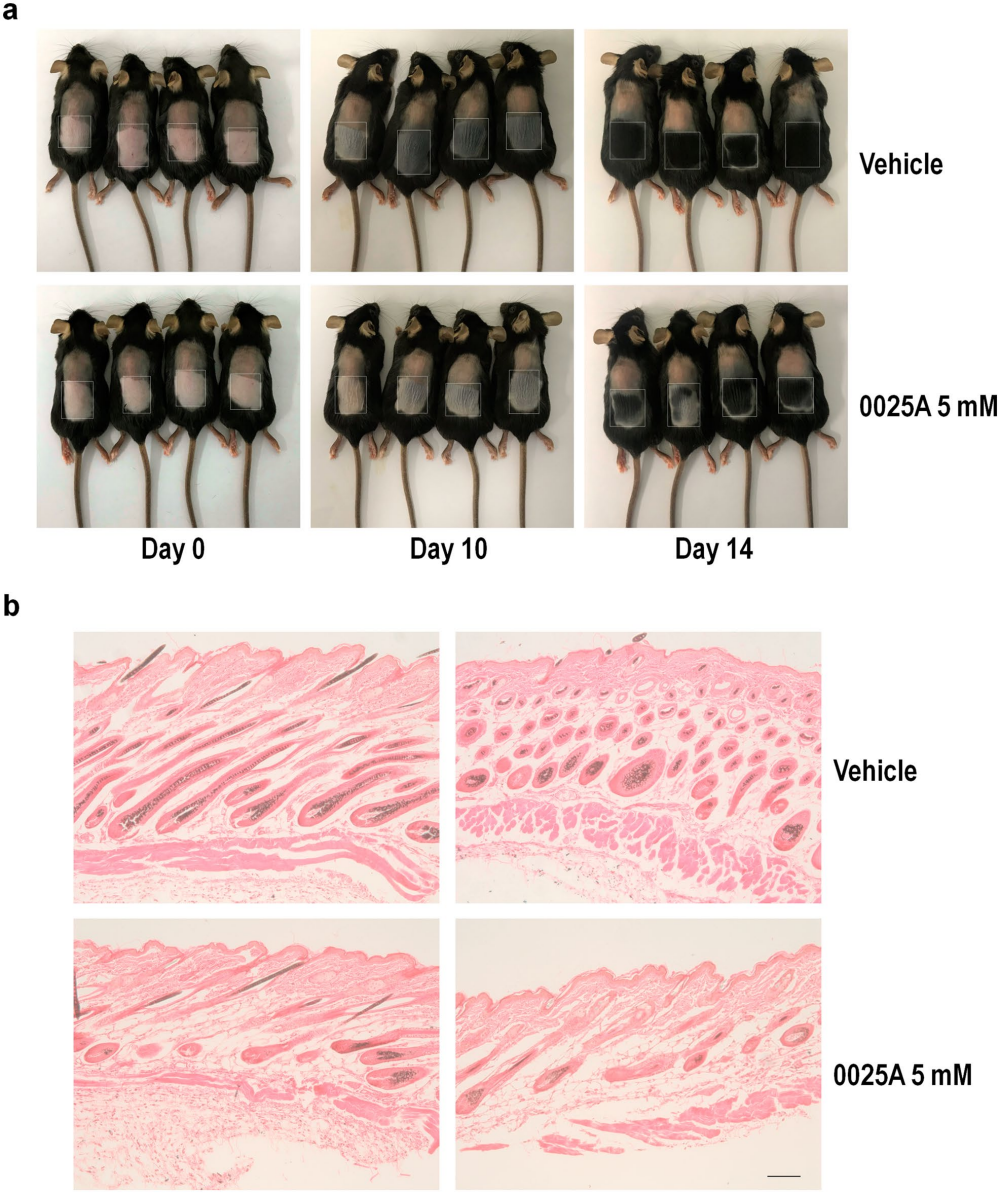
0025A inhibits Hh-dependent hair growth and hair follicle morphogenesis after depilation. **a** Eight-week old female C57BL/6 mice were shaved. Then the bottom half of dorsal region were depilated with Nair and treated once daily with 30 μL of vehicle control (95% acetone/5% DMSO) or 5 mM Smo antagonist 0025A for 14 consecutive days. The photos were taken on 0 Day, 10 Days and 14 Days after depilation. The dotted boxes represented the depilated area. **b** Hematoxylin and eosin staining on dorsal skin sections (vehicle and 0025A, n = 3). Scale bar, 10 µm

Reports have demonstrated that Shh signaling is closely related to regulate hair follicle morphogenesis [5, 40, 41]. To confirm the inhibitory effect of 0025A on Shh signaling in vivo, we shaved the dorsal hair of 8 week female C57BL/6 mice and depilated with Nair, which could induce hair regrowth into anagen phase of the hair follicles. After depilation, we treated topical skin areas with vehicle and 0025A once daily for 14 consecutive days and observed the pigment formation and hair regrowth. The photographs were taken at 10 and 14 days. We found that skin became more gray (hair) in vehicle treated mice on 10 days. In addition, most of hair in vehicle treated mice grew back on 14 days. However, hair growth was largely arrested in 0025A treated group (Fig. 9a). Consistent with the gross phenotype, the histological analysis indicated that the pigment formation and hair follicle morphogenesis was blocked in 0025A treated group (Fig. 9b). Besides, we also observed 0025A inhibited the expression of Gli1 in hair follicles in the dorsal skin sections by the immunostaining of Gli1 (Additional file 2: Fig. S2). These results demonstrated that 0025A could suppress Shh signaling mediated hair growth in vivo.

## Discussion

Abundant evidences demonstrate that excessive Hh signaling induce the development of many cancers such as the advanced BCC and MB. The seven-transmembrane protein Smo is the main transducer of Hh signaling pathway and can be used as an important drug target. Therefore, many Smo inhibitors have been under investigation in clinical trials. Among them, GDC-0449 (vismodegib) and LDE-225 (sonidegib) were approved by FDA for treatment of advanced BCC in 2012 and 2015, respectively. As we known, acquired drug resistance is the main obstacle of first generation of Smo inhibitors. According to the clinical trials, GDC-0449 and LDE-225 emerged drug resistance because of the D473H mutation in Smo [27, 39]. Thus far, there are no available drugs for treatment of Hh-related diseases, which inspires a surge of Smo inhibitor researches.

In this study, we made use of the Smo/βarr2-GFP high-throughput assay platform, and screened Smo inhibitors in our propriety small molecules library with the concentration of 5 μM. Through this effort, we discovered several active hits under screening including 0025A. In the Smo/βarr2-GFP internalization assay, we found 0025A was the most effective antagonist and be able to inhibit the cellular puncta of βarr2-GFP at the nano level. In addition, when we used Shh-CM or SAG to enhance Gli1 transcription, we observed that 0025A can act as a potent inhibitor and dose-dependently inhibit target gene Gli1 expression. At the same time, we have confirmed that 0025A would act on Smo receptor to inhibit Hh signaling pathway. Moreover, this lead compound also could interact with mutated Smo-D473H in contrast to GDC-0449 demonstrated by using Bodipy-cyclopamine competition binding assay. 0025A will provide a new tool to study the Smo-D473H mediated drug resistance and a candidate to treat the drug resistance to improve the cancer therapeutic success.

In addition, reports have demonstrated that Hh signaling is closely related to regulate hair follicle morphogenesis. In this study, we revealed that 0025A could inhibit the pigment formation, hair follicle morphogenesis and Hh-induced hair growth after depilation, further proving the inhibition of 0025A on Hh signaling pathway and indicating that 0025A could penetrate the skin barrier and might be suitable for the treatment of BCC. Next, the therapeutic effect of 0025A on BCC and MB will be further studied in vitro and in vivo. These studies would provide basis for future clinical trials and may provide a new therapy for patients with cancers resulted from abnormal Hh activities.

## Conclusion

In summary, we identify 0025A as a potent Smo antagonist of Hh signaling by using the Smo/βarr2-GFP high-throughput screening platform. In this study, we show that 0025A is capable of interacting with wild-type Smo or mutant Smo-D473H and reduce the accumulation of Smo on primary cilia and the expression of Gli upon Hh stimulation. These findings not only provide a new approach to study the insights into mechanisms underlying drug resistance in refractory cancers, but also layout a foundation for developing second generation of Smo inhibitor therapy.

## Materials and methods

### Chemicals

0025A was synthesized by Bellen. Smo agonist (SAG) was synthesized by YuezhiKangtai Biomedicines (Beijing, China). GDC-0449 and Cyclopamine were purchased from Cayman. Bodipy-cyclopamine was purchased from Biovision.

### *N-Shh* conditioned medium

*N-Shh* conditioned medium was prepared by transfection of pRK5-ShhN plasmid into 293FT cells. The medium was harvested at 24 h and 48 h after transfection, pooled and centrifuged at 1000 rpm for 10 min. The supernatant was stored at 4 °C until used.

### Cell culture, plasmids, and western blotting

293FT, NIH3T3, U2OS and PTCH^−/−^ MEF cells were cultured in DMEM (Gibco) supplemented with 10% fetal bovine serum (Hyclone). βarr2-GFP, WT Smo and Smo-633 mutant have been previously described [31]. For standard assay of 0025A effect on Gli1 expression, NIH3T3 cells were plated in 12-well plate at 2 × 10^5^ per well for overnight. On the next day, the cells were starved in DMEM containing 0.5% FBS for 1 h at 100% confluence, then stimulated with 20% Shh-CM or 100 nM SAG without or with different concentration of 0025A. 24 h later, cells were lysed with cell lysis buffer consisting of 50 mM Tris–HCl, PH 7.4, 100 mM NaCl, 2 mM EDTA, 1% NP40, protease inhibitors, and centrifuged with at 12,000 rpm for 10 min at 4 ℃. Cell lysates were collected and immunoblotted with following antibodies: Gli1 (CST, 2534s, 1:1000); Tubulin (CST, 3873s, 1:3000).

### Immunostaining

For tissue staining, dorsal skin were fixed with 4% paraformaldehyde, dehydrated in graded ethanol series and embedded in paraffin. Sections of 5 µm thickness were prepared by Leica RM2255 rotary microtome and used for immunostaining after deparaffinization. For cell staining, Ptch^−/−^ MEF cells cultured on cover-slips were fixed with 4% paraformaldehyde, blocked with 5% BSA for 1 h and incubated overnight with primary antibody. The following antibodies were used for immunostaining: Smoothened (Santa Cruz, sc-166685, 1:100); ARL 13B (Proteintech, 17711-1-AP, 1:100); Gli1 (Proteintech, 66905–1-Ig, 1:200). The secondary antibodies conjugated with Alexa Fluor 488 or 568 were purchased from Invitrogen, those conjugated with horse radish peroxidase (HRP) using DAB as chromogen from Cwbio. Sections were photographed by LSM710 Zeiss confocal microscope and analyzed with Image J software.

### RNA isolation, reverse transcription and real-time PCR

Cells were harvested and extracted total RNA using the TRIzol reagent (Thermo), and reverse transcription was performed with PrimeScript™ RT reagent Kit (TaKaRa). Real-time PCR was carried out according to the protocol of the manufactory. The primers for real-time PCR were mouse Gli1, F: 5ʹ-CTCAAACTGCCCAGCTTAACC.

C-3ʹ, R: 5ʹ-TGCGGCTGACTG TGTAAGCAGA-3ʹ; Actin, F: 5ʹ-GCAAGTGCTTC.

TAGGCGGAC-3ʹ, R: 5ʹ-AAGAAAGGGTGTAAAACGCAGC-3ʹ.

### Construction of Smo D473H mutagenesis

Flag Smo-D473H point mutation was established by using Fast Mutagenesis System (TransGen Biotech). Primers were as follows: F: 5ʹ-AGCTGCCACTTCTAC.

CACTTCTTCAA-3ʹ, R: 5ʹ-GGTAGAAGTGGCAGCTGAAGGTAATG-3ʹ.

### Bodipy-cyclopamine binding assay

Human Flag Smo WT or Smo mutant (D473H) was transfected in HEK293 cells. 24 h later, the HEK293 cells were trypsinized, washed in phenol-red free DMEM supplemented with 0.5% fetal bovine serum, fixed with 4% paraformaldehyde for 10 min at room temperature and incubated with 5 nM Bodipy-cyclopamine and different concentration of indicated compounds for 2 h at 37 ℃. Then the treated cells were centrifuged and the fluorescent signals were analyzed by flow cytometry.

### Animal studies

The animal studies were performed as previously described [30]. Briefly, female C57BL/6 mice (aged 7 to 8 weeks) were shaved dorsal hair and the bottom half of shaved area was depilated with Nair for 2 min. After depilation, the mice were topically treated with 30 μL of 5 mM 0025A dissolved in a vehicle of 95% acetone/5% DMSO or the vehicle once daily for 14 consecutive days and observed the pigment formation and hair regrowth. During the experimental procedures, the mice were anesthetized by 3% isoflurane and the studies were performed according to the ethical principles of animal welfare of Beijing Institute of Neurosurgery.

### Statistical analysis

All statistical analyses were acquired from at least three independent experiments, and values were expressed as means ± SEM, and two-sample comparisons were performed using an unpaired Student’s *t* test. *P < 0.05 was considered as statistical significance. All statistical analyses were presented using Prism software (GraphPad Software, Inc, CA).

## Supplementary Information

**Additional file 1: Fig. S1.** 0025A inhibits the expression of Ptch1 induced by Shh-CM. **a** Serum-starved NIH3T3 cells were treated with Shh-CM for 24 h. Cells were analyzed for mRNA levels of Ptch1. **b** Serum-starved NIH3T3 cells were treated by Shh-CM with vehicle, different concentration of 0025A, or 1 μM GDC-0449 (GDC-1) for 24 h. Cells were analyzed for mRNA levels of Ptch1. All data are means ± SEM (student’s t-test). *P < 0.05, **P < 0.01, ns, not significant.

**Additional file 2: Fig. S2.** 0025A inhibits the expression of Gli1 in hair follicle. Dorsal skin section samples treated with vehicle or 0025A were stained with anti-Gli1 antibody for Gli1 expression. Scale bar, 10 µm.

## Data Availability

All data generated or analyzed during this study are included in this published article.

## Abbreviations

Hh: Hedgehog
Ptch1: Patched1
Smo: Smoothened
BCC: Basal cell carcinoma
MB: Medulloblastoma
βarr2-GFP: βArrestin2-GFP
Shh: Sonic hedgehog
Shh-CM: N-*Shh* conditioned medium
Cyc: Cyclopamine
