# Supplementary figures and images for: Identification of a potent antagonist of smoothened in hedgehog signaling
Source: Cell Biosci. 2021 Mar 2;11:46. doi: 10.1186/s13578-021-00558-9 (PMC7923671; doi:10.1186/s13578-021-00558-9)

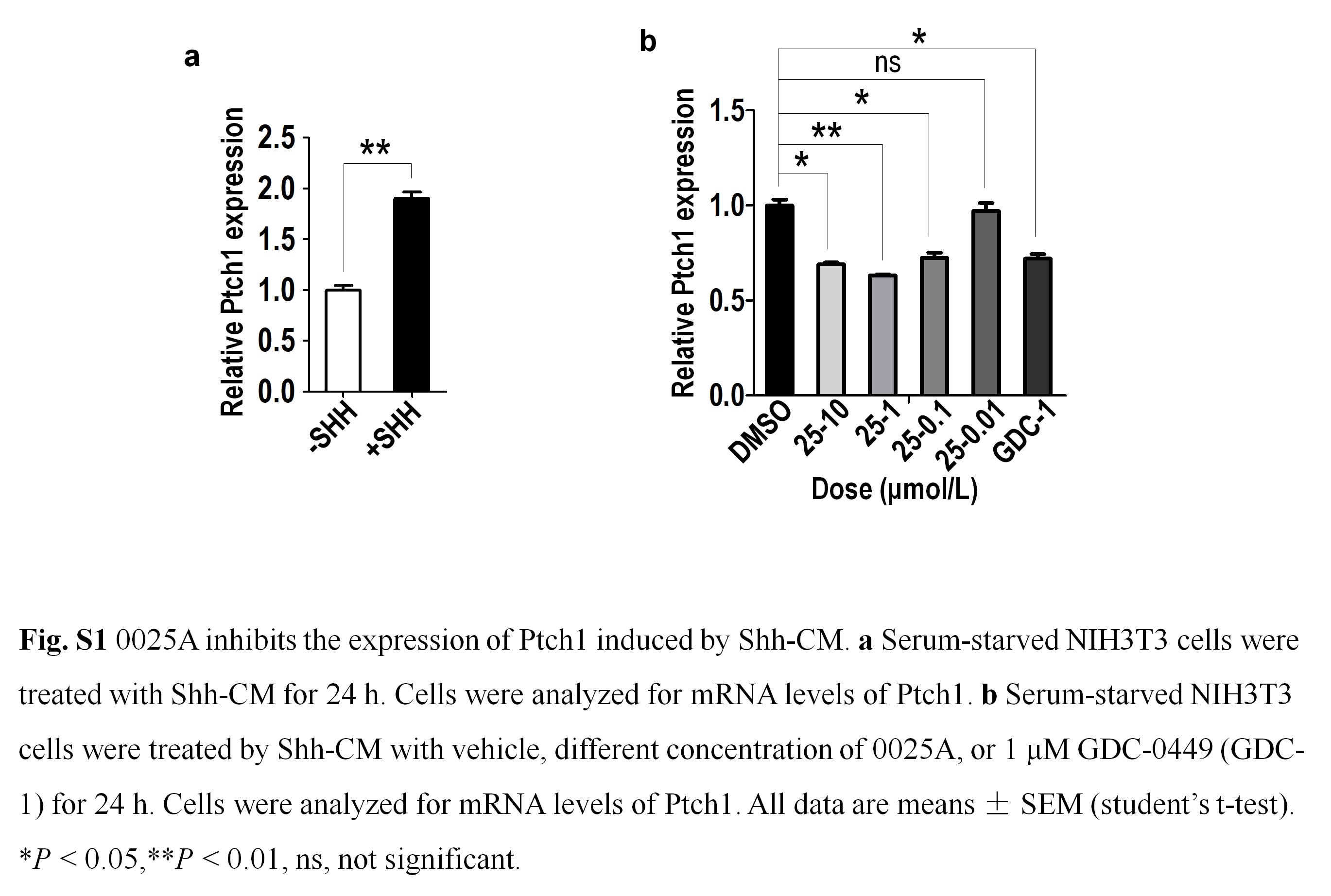

Supplement: Supplementary file 1 — Additional file 1: Fig. S1. 0025A inhibits the expression of Ptch1 induced by Shh-CM. a Serum-starved NIH3T3 cells were treated with Shh-CM for 24 h. Cells were analyzed for mRNA levels of Ptch1. b Serum-starved NIH3T3 cells were treated by Shh-CM with vehicle, different concentration of 0025A, or 1 μM GDC-0449 (GDC-1) for 24 h. Cells were analyzed for mRNA levels of Ptch1. All data are means ± SEM (student’s t-test). *P < 0.05, **P < 0.01, ns, not significant. [file 13578_2021_558_MOESM1_ESM.tif]

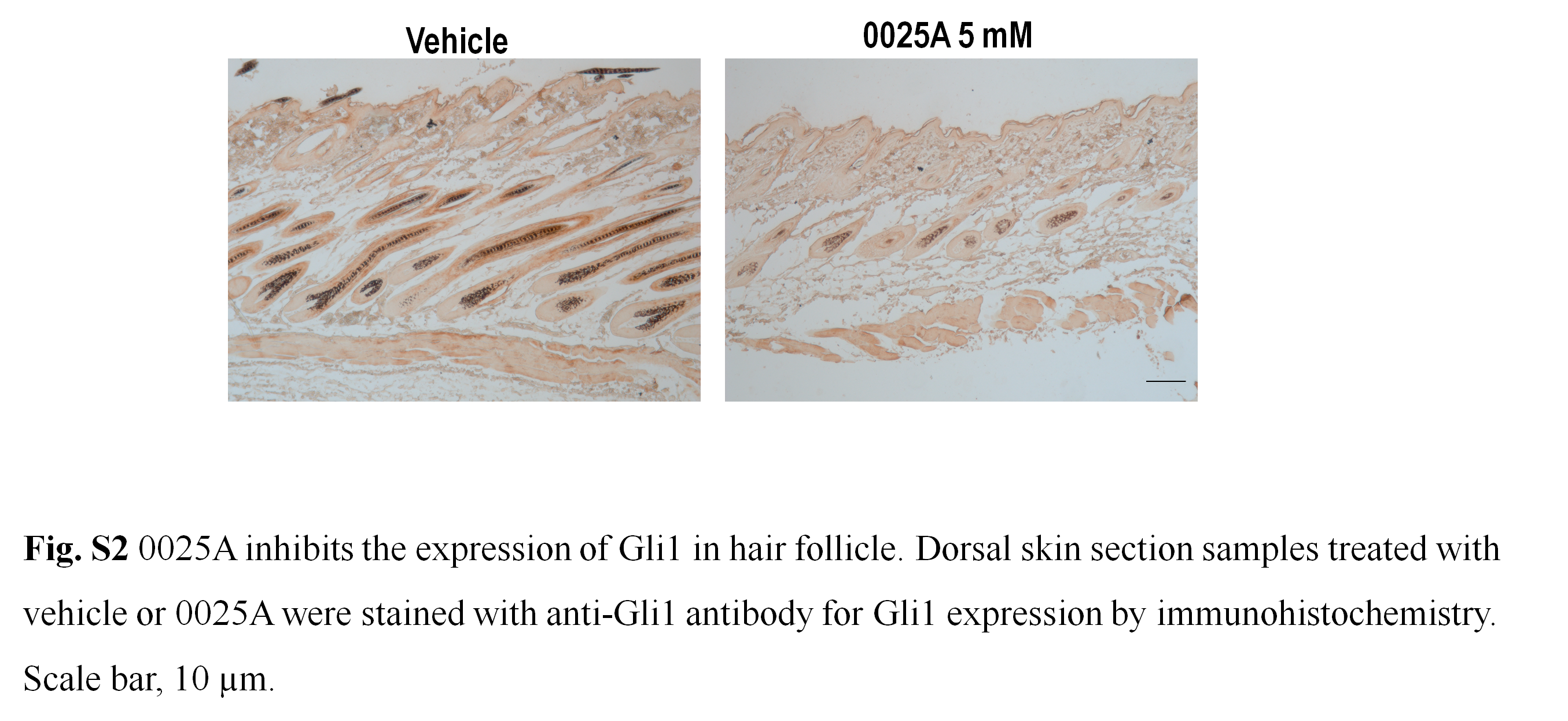

Supplement: Supplementary file 2 — Additional file 2: Fig. S2. 0025A inhibits the expression of Gli1 in hair follicle. Dorsal skin section samples treated with vehicle or 0025A were stained with anti-Gli1 antibody for Gli1 expression. Scale bar, 10 µm. [file 13578_2021_558_MOESM2_ESM.tif]
